# MicroRNA Expression Profiling of Human Respiratory Epithelium Affected by Invasive Candida Infection

**DOI:** 10.1371/journal.pone.0136454

**Published:** 2015-08-27

**Authors:** Syed Aun Muhammad, Nighat Fatima, Nawazish-i-Husain Syed, Xiaogang Wu, X. Frank Yang, Jake Y. Chen

**Affiliations:** 1 Institute of Molecular Biology and Biotechnology, Bahauddin Zakariya University (BZU), Multan, Pakistan; 2 Department of Pharmacy, COMSATS Institute of Information Technology, Abbottabad, 22060, Pakistan; 3 University College of Pharmacy, University of the Punjab, Lahore, Pakistan; 4 Institute for Systems Biology (ISB), Seattle, WA, United States of America; 5 Department of Microbiology and Immunology, Indiana University School of Medicine, Indianapolis, IN, United States of America; 6 Indiana Center for Systems Biology and Personalized Medicine, Indiana University-Purdue University, Indianapolis, IN, United States of America; 7 School of Informatics and Computing, Indiana University, Indianapolis, IN, United States of America; 8 Department of Computer and Information Science, School of Science Purdue University, Indianapolis, IN, United States of America; 9 Institute of Biopharmaceutical Informatics and Technology, Wenzhou Medical University, Wenzhou, Zhejiang Province, China; Sabanci University, TURKEY

## Abstract

Invasive candidiasis is potentially life-threatening systemic fungal infection caused by *Candida albicans* (*C*. *albicans*). Candida enters the blood stream and disseminate throughout the body and it is often observed in hospitalized patients, immunocompromised individuals or those with chronic diseases. This infection is opportunistic and risk starts with the colonization of *C*. *albicans* on mucocutaneous surfaces and respiratory epithelium. MicroRNAs (miRNAs) are small non-coding RNAs which are involved in the regulation of virtually every cellular process. They regulate and control the levels of mRNA stability and post-transcriptional gene expression. Aberrant expression of miRNAs has been associated in many disease states, and miRNA-based therapies are in progress. In this study, we investigated possible variations of miRNA expression profiles of respiratory epithelial cells infected by invasive Candida species. For this purpose, respiratory epithelial tissues of infected individuals from hospital laboratory were accessed before their treatment. Invasive Candida infection was confirmed by isolation of *Candia albicans* from the blood cultures of the same infected individuals. The purity of epithelial tissues was assessed by flow cytometry (FACSCalibur cytometer; BD Biosciences, Heidelberg, Germany) using statin antibody (S-44). TaqMan quantitative real-time PCR (in a TaqMan Low Density Array format) was used for miRNA expression profiling. MiRNAs investigated, the levels of expression of 55 miRNA were significantly altered in infected tissues. Some miRNAs showed dramatic increase (miR-16-1) or decrease of expression (miR-17-3p) as compared to control. Gene ontology enrichment analysis of these miRNA-targeted genes suggests that Candidal infection affect many important biological pathways. In summary, disturbance in miRNA expression levels indicated the change in cascade of pathological processes and the regulation of respiratory epithelial functions following invasive Candidal infection. These findings contribute to our understanding of host cell response to Candidal systemic infections.

## Introduction

*Candida albicans* (*C*. *albicans*) is the causative agent of candidiasis in humans that causes superficial as well as invasive (systemic) infection [1]. The symptoms may vary and depending upon the infected area and ranges from minor complications to potentially life-threatening conditions [2].

The risk of candidiasis begins with colonization of *C*. *albicans* on the surfaces of mucocutaneous membranes as well as the lining of the respiratory epithelium. The systemic candidiasis with the rise of *C*. *albicans* level in the bloodstream is most prevalent in fungal infected tissues; this menace has been increased in immunocompromised individuals [3]. Following Candidal infection, injury of epithelium can result in damage of structural integrity and eventually loss of physiological functions [4] and development of fatal lung diseases [5]. Fungal spores can destruct sensitive lung tissues which lead to scar formation. Regulation of these physiological processes requires complex progressive modifications in epithelial cell biology, which is largely controlled by the expression of genetic elements [6]. The expression of small non-coding RNA molecules termed miRNAs is involved to coordinate regulation of expression [7] of at least 30% of human genes. Thus, miRNAs are now considered as master regulators of gene expressions [8].

MiRNAs have been shown to play an important role in epithelial cell physiology. The expression of Dicer in airway epithelium, the enzyme complex responsible for catalyzing miRNA precursors, is crucial for structural morphogenesis and tissue development [9]. Therefore, epithelial cell injury after fungal infection could lead to aberrant expression of miRNA, resulting loss of physiological functions.

Recent reports have revealed significant variations in miRNAs during lung diseases. For example, it has been shown that miR-21 has critical role in pathophysiology of lungs [10]. Similarly in some other experimental analysis, mice lacking miR-155 showed autoimmune phenotypes in the lungs with increased leukocyte invasion [11, 12].

Despite that a number of studies showed the role of miRNAs in disease development [13–15], their influence on the regulation of gene expression involved in invasive candidiasis remain unclear. In addition, comprehensive studies on miRNA association in epithelial damage, inflammation, asthma and the pathogenesis of airway remodeling are lacking.

Upon pathogen infection, variations in the host cell miRNA profile may either show a cellular defense mechanism or a subversion approach developed by the pathogen. It is possible that Candida species may manipulate the miRNA genetic network of infected host cell. Therefore, we hypothesized that invasive Candida infection may change the miRNA expression profile during the progression of disease.

The purpose of this study was to investigate the possible involvement of miRNAs by probing their expression profile in epithelial tissues. We profiled a set of 265 miRNAs of four individuals. The results showed that invasive candidiasis modifies the expression of about 20% of miRNAs examined. This study may potentially lead to a novel therapeutic approach for combating pain, inflammation, asthma, lung cancer and invasive candidiasis.

## Materials and Methods

### Sample collection

Respiratory epithelial tissues and blood samples from four hospitalized patients diagnosed with invasive candidiasis were collected from local hospital laboratory (Nishtar Hospital). The individuals in this study have given written informed consent to report their samples for research purpose. The average age of all four patients was 40 years (three males and one female). Four controls were included in this study. All samples were immediately frozen and stored at -80°C. The study protocol was reviewed and approved by the departmental ethical committee of Institute of Molecular Biology and Biotechnology, BZU Multan.

### Isolation of *C*. *albicans* in blood samples

*C*. *albicans* was directly isolated from blood samples of infected individuals using selective and differential chromogenic medium (CHROMagar Candida) according to the method described by Horvath *et al*. in 2003 [16]. Aliquot of blood culture was taken and plated onto CHROMagar Candida media. The plates were incubated at 35°C and typical appearance of microorganisms was observed.

### Assessment of cell purity and viability

The purity and viability of epithelial cells was assessed by flow cytometry (FACSCalibur cytometer; BD Biosciences, Heidelberg, Germany) using statin antibody (S-44). Before staining procedure, the cells were incubated for 30 minutes with 10% FCS to avoid nonspecific binding to antibodies. At least 500 cells were counted for assessment. The epithelial cells were suspended in phosphate buffered saline (PBS) and fraction of these isolates were placed on glass slides and left to dry at 25°C for 30 minutes. The cell smears were then fixed by incubation in organic solvents (50% acetone and 50% methanol) for 8 minutes at room temperature. The fractions fixed on glass slides were then rinsed with PBS and directly probed with S-44 monoclonal antibody as previously described [17].

### RNA isolation

RNA isolation from infected and normal samples was performed with the use of an Exiqon RNA isolation kit according the manufacturer’s instructions. Samples were frozen at −70°C for subsequent use in microarray experiments. The concentration of total RNA was determined using NanoDrop ND-1000 spectrophotometer, and the quality and integrity was assessed by using Lab901 Gene Tools System.

### Micro RNA profiling

The miRNA expression profiling of human respiratory epithelial cells was performed by real-time PCR in three replicates using microfluidic cards based on Sanger miRBase 9.2 (TaqMan Array Human MicroRNA Card A and B, Applied Biosystems) according to manufacturer’s recommendations. PCR amplification was performed using TaqMan miRNA reverse transcription kit and Megaplex RT Primers (Megaplex RT-primers) and the resultant cDNA combined with TaqMan Universal PCR Master Mix (Applied Biosystems), AmpErase UNG was loaded into the arrays and real-time PCR was performed according to the manufacturer’s protocol. SDS 2.3 software was used for collecting raw data. The comparative C(T) analysis was performed for relative quantification of gene expression after ignoring outliers by using Grubbs-outlier test [18]. The abundance of each miRNA was normalized to the geometric average of the 2 endogenous controls (U6 snRNA-001973) according to the previous report [19]. The DDCt is one of the methods used to calculate real–time PCR results [20] and it was calculated as the difference between infected and non-infected DDCt where the mean of endogenous control was used to normalize the Ct value of sample. The results are expressed in fold change, and the undetectable values are indicated as ND (not detectable).

### Cluster and statistical analyses

The hierarchical clustering method of miRNA after expression profiling was analyzed using web-available “Cluster and TreeView” (http://genome-www5.stanford.edu/MicroArray/SMD/restech.html). The clusters were ordered based on number of genes, and profiles were ordered by significance (p-value) using this software.

### Gene Ontology (GO) and pathways analysis

To identify potential common biological pathways for miRNAs showing similar expression profiles in cluster analysis, we performed pathway enrichment analysis. GO analysis was performed using public available web-based tool David (database for annotation, visualization, and integrated discovery) [21] and KEGG pathways [22]. An enrichment interaction analysis was performed using Cytoscape v3.1.1 [23] based on the GO terms. Overrepresented biological processes were selected at the threshold p-Value ≤ 0.05 and minimum gene counts belonging to an annotation term ≥ 2%.

## Results

### Confirmation of *C*. *albicans* in blood samples

**Fig 1:**
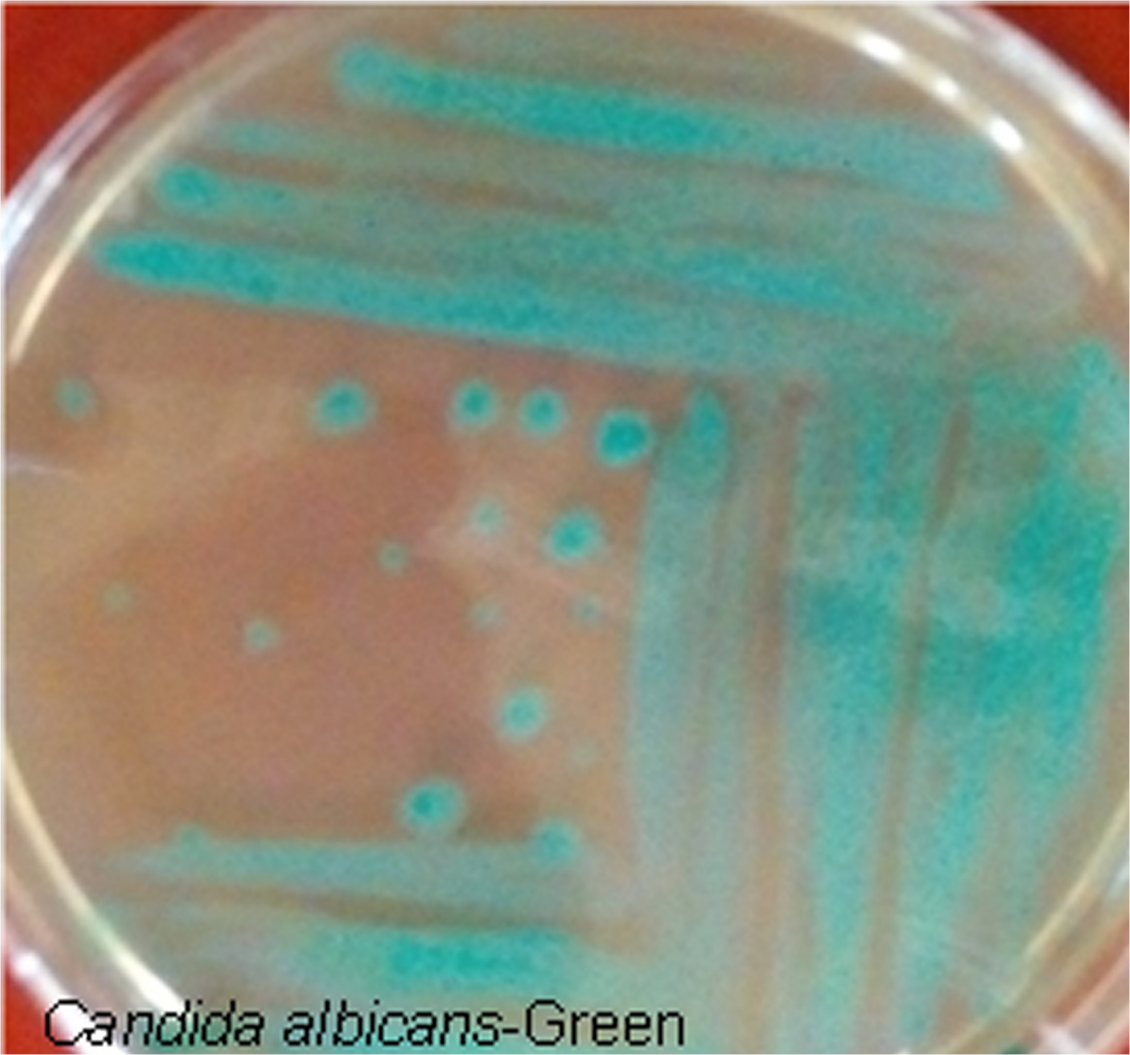
Selective and differential chromogenic media were used for isolation and differentiation of Candida species. Green color appearance on media indicates the presence of *Candida albicans*.

*C*. *albicans* in blood samples of infected individuals was directly isolated using differential chromogenic medium. This medium is used selectively for isolation of Candida species based on the morphological appearance of this fungus on this medium (changes to green color) (Fig 1).

### Flow cytometry analysis of isolated respiratory epithelial cells

**Fig 2:**
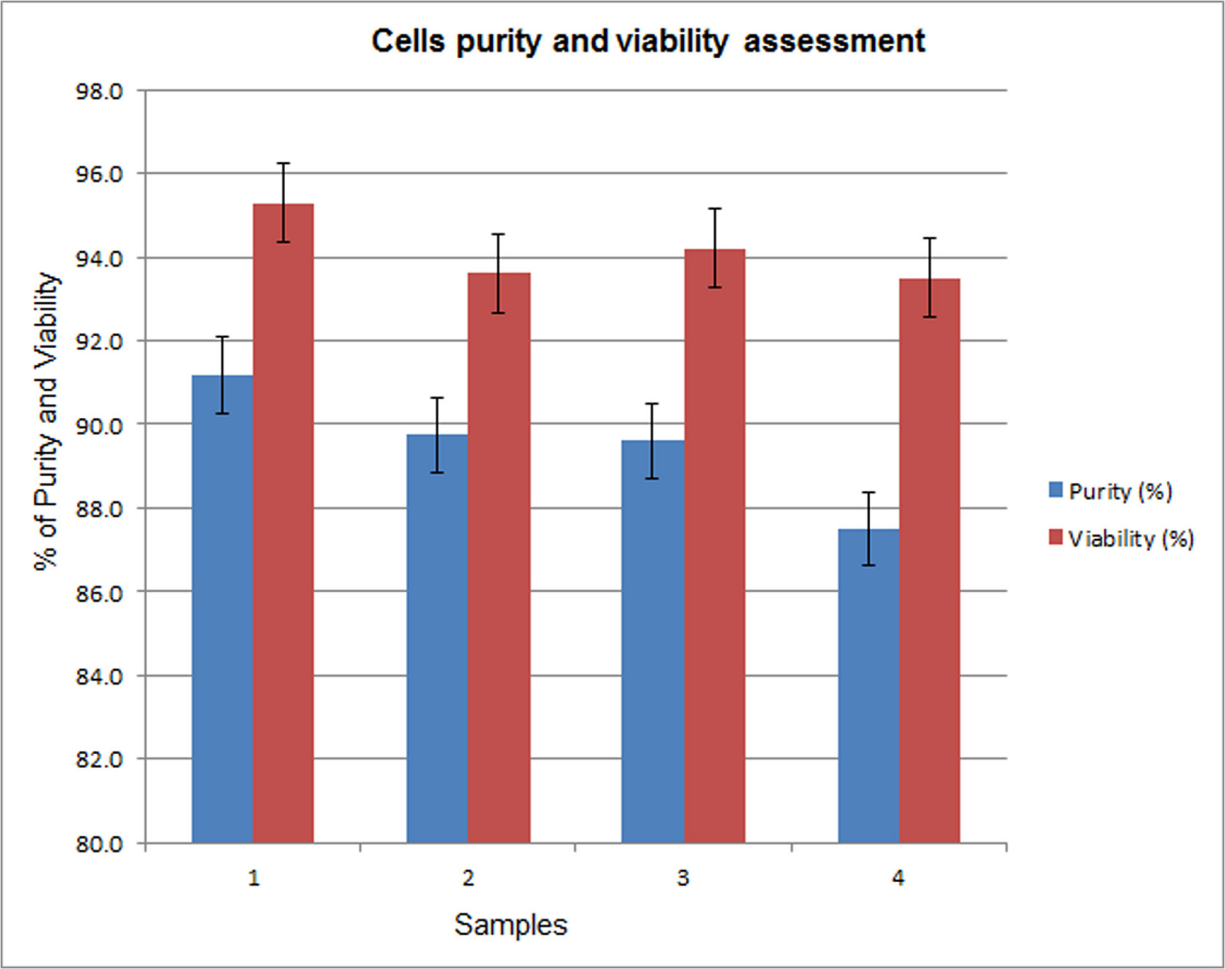
Epithelial cell purity and viability assessment. It represents the ratio of total statin stained cells to total cells determined by nuclear staining. A total of 500 cells were analyzed. Data represent the mean ± standard deviation.

The purity of isolated cell populations was assessed to ensure the sample quality for contamination. The cells were probed and analyzed by Flow cytometry using S-44 antibody to determine the percentage of the human respiratory epithelial cell (hREC) population infected by *Candida albicans*. The purity and viability of the preparations was over 85% and 94% respectively (Fig 2).

### miRNA expression Profiling

**Fig 3:**
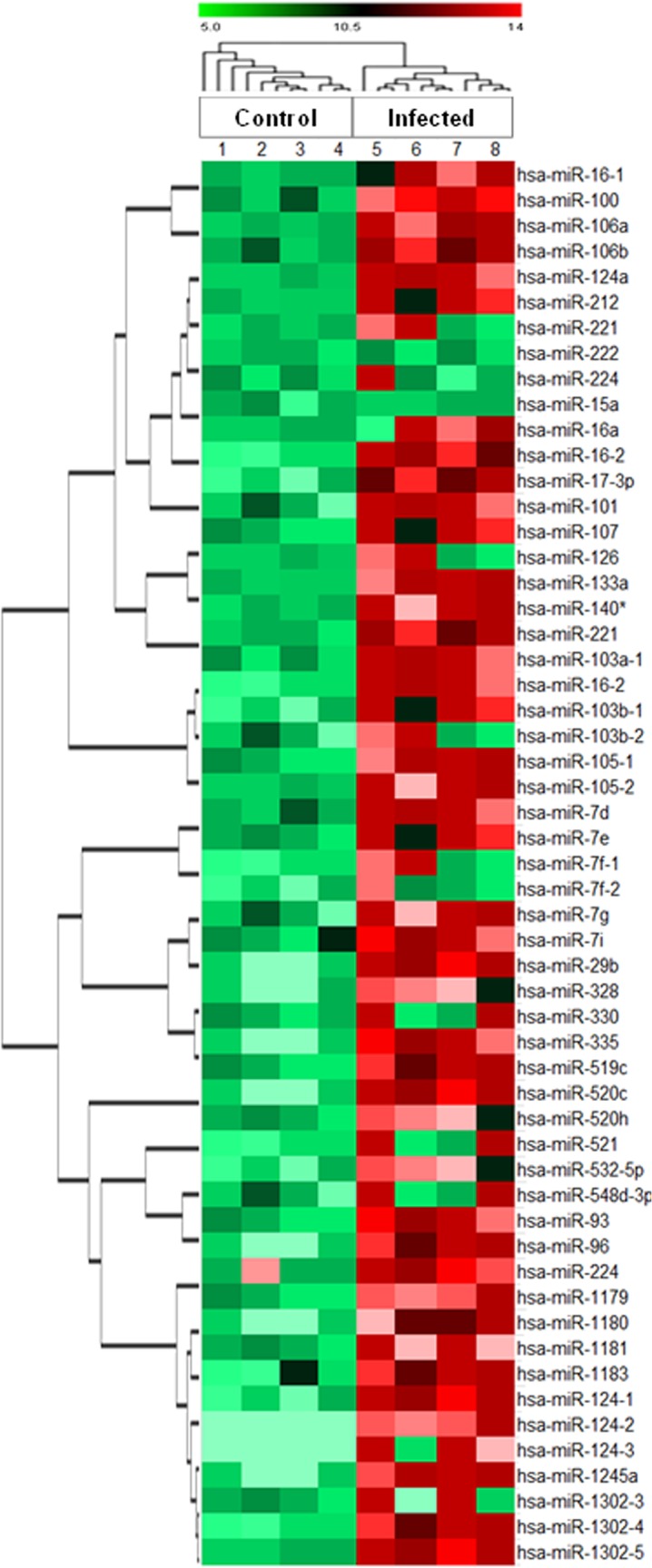
Hierarchical cluster analysis of aberrant miRNAs expression in human respiratory epithelium infected with *Candida albicans*. This figure contains a “heat map” containing colors (red, green, and black), with dendrograms. The rows represent genes and color represents the expression level of the gene.

**Fig 4:**
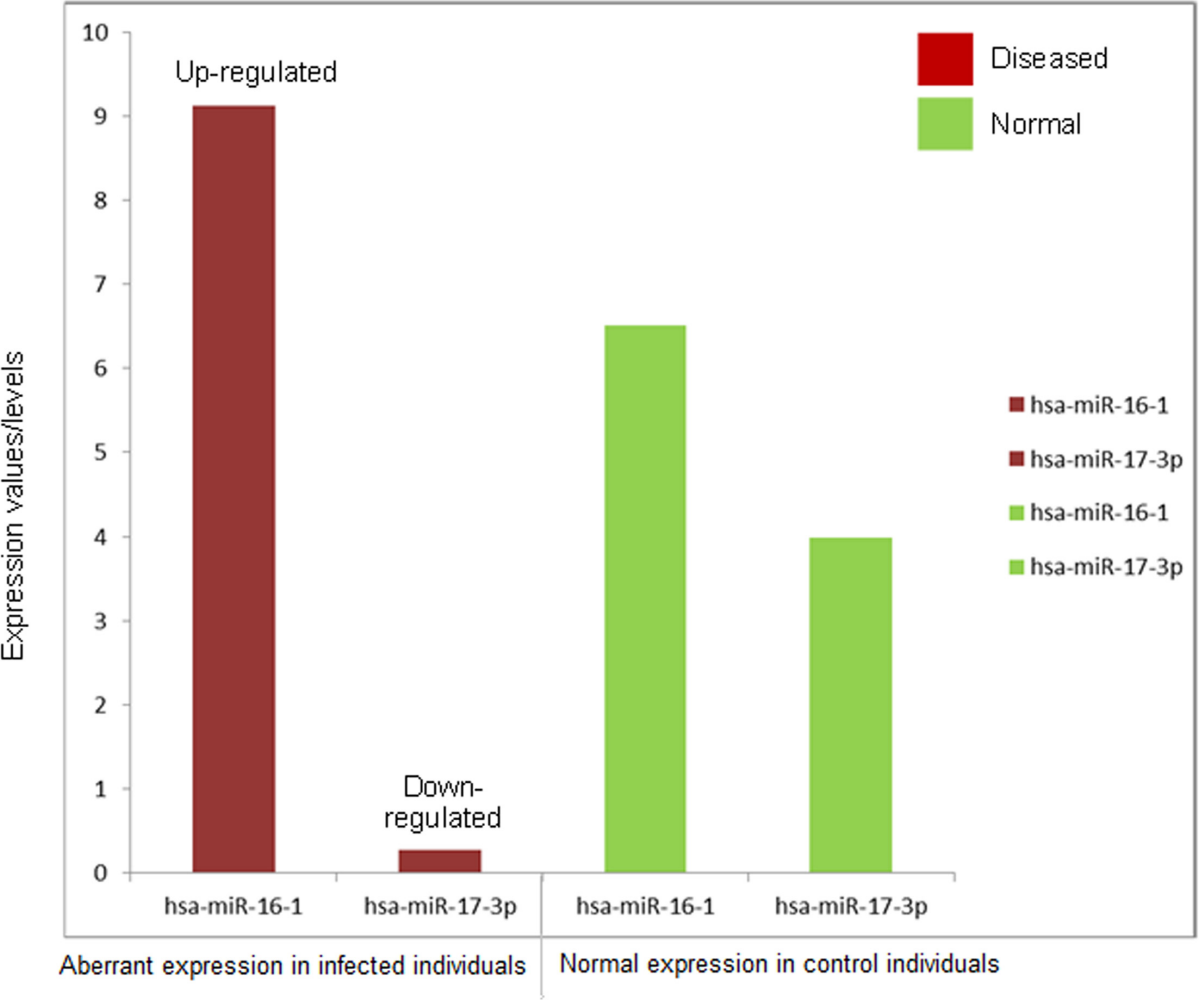
Aberrant expression of hsa-miR-16-1 and hsa-miR-155 (the most significant genes based on fold change and p-values). hsa-miR-16-1 is up-regulated while hsa-miR-155 miRNA is down-regulated in individuals infected with invasive candidiasis as compared to normal expression levels (control).

Expression profiling study showed 55 mature miRNAs that were expressed differently after invasive candidiasis with a fold change above 2.0. Among 55 aberrant miRNAs, 26 were down-regulated as compared to normal expression. Hierarchical clustering of these 55 aberrantly expressed miRNAs of four infected individuals has been shown in Fig 3. The cluster with dendrograms presented qualitative means of assessing the similarity and variations between genes of control and diseased samples. We only included miRNAs that revealed consistent variations in samples with fixed cut-off values. Based on the p-statistics and fold change values, eighteen miRNAs were found to undergo significant variations (Table 1). As compared to normal expression in control individuals, the aberrant expression of hsa-miR-16-1 and hsa-miR-17-3p (the most significant genes in this study) has been shown in Fig 4.

**Table 1:** List of selected most significant up and down-regulated expression miRNAs in invasive candidiasis.

**Up-regulated Expression**			
**S. No.**	**miRNA**	**Fold change**	**P-value**
1	hsa-miR-16-1	9.0001531	0.0110932
2	hsa-miR-100	4.1092773	0.0101691
3	hsa-miR-106a	12.87887	0.0300142
4	hsa-miR-106b	7.3201244	0.0397011
5	hsa-miR-124a	3.6494651	0.0051253
6	hsa-miR-212	3.1439637	0.0107327
7	hsa-miR-221	3.290284	0.0037317
8	hsa-miR-222	2.5721577	0.0405053
9	hsa-miR-224	9.0601039	0.0210822
**Down-regulated Expression**			
**S. No.**	**miRNA**	**Fold change**	**P-value**
1	hsa-miR-15a	0.3607081	0.0181974
2	hsa-miR-16a	0.4889374	0.0128966
3	hsa-mir-16-2	0.5970402	3.813E − 04
4	hsa-miR-17-3p	0.278258	0.032224
5	hsa-miR-101	0.5970007	3.613E − 06
6	hsa-miR-107	0.2584031	0.0433912
7	hsa-miR-126	0.5783737	0.000837
8	hsa-miR-133a	0.3634912	0.0189611
9	hsa-miR-140*	0.3225766	0.0005821

### QRT-PCR validation study

**Fig 5:**
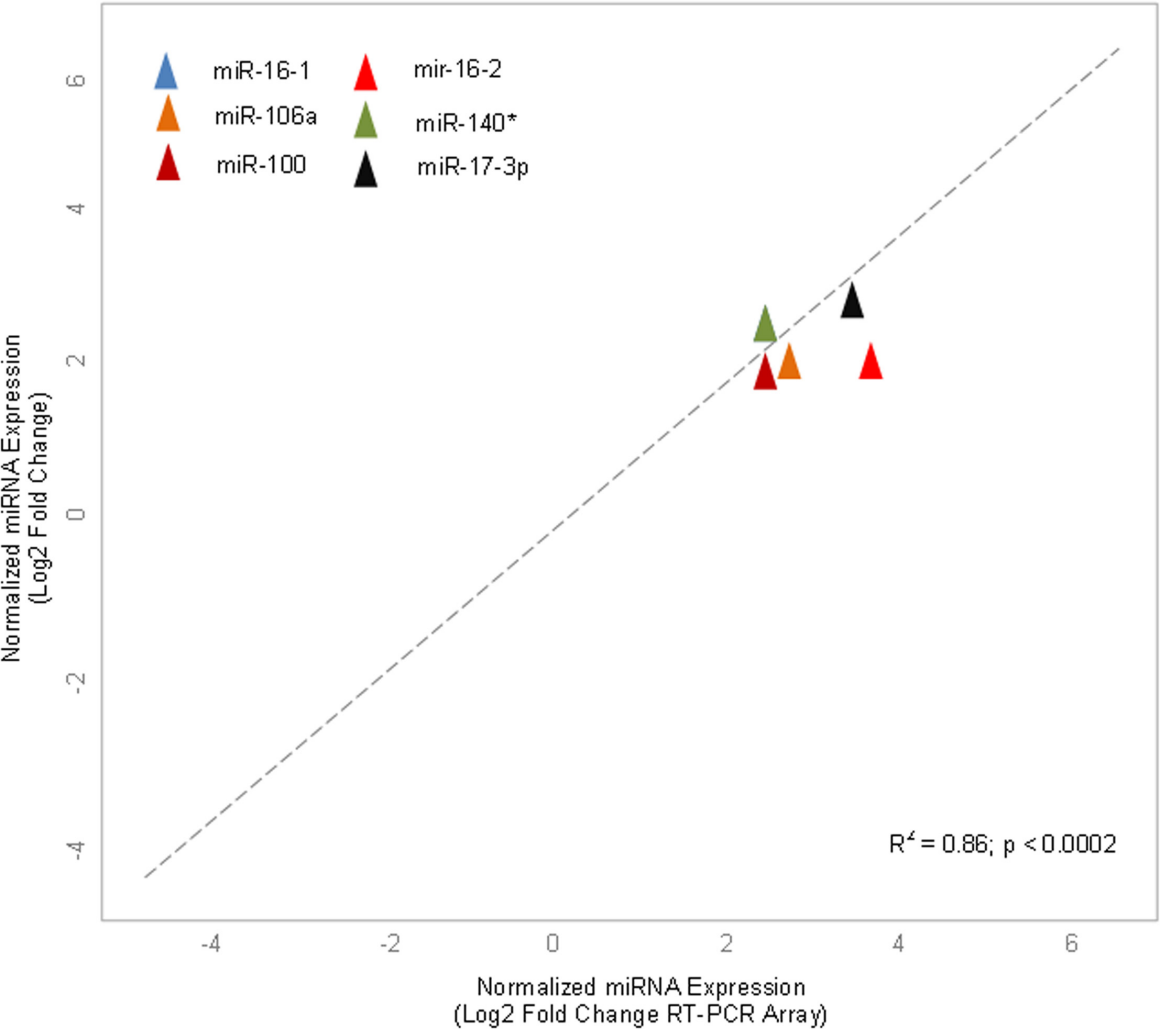
qRT-PCR array validation of the randomly selected miRNAs. Plot graph shows correlation between expression levels of miRNAs measured by array analysis and expression levels measured by individual qRT-PCR. −ΔΔCt method was applied for this analysis.

A qRT-PCR analysis was performed on randomly selected six miRNAs (miR-16-1, 106a, 16–2, 100, 140*, 17-3p) from 55 differentially expressed miRNAs to validate accuracy of array-generated data. It was observed that the data generated by PCR-array revealed stable results as produced by qRT-PCR (Fig 5). The correlation-coefficient between the mean values of the four individuals generated by both techniques for each miRNA was statistically significant (r^2^ = 0.86; p < 0.0002).

### Cluster and statistical analysis

Each miRNA expression profile passed the filtering criteria when tested by a clustering algorithm. During the infection, miRNAs expression levels were modulated. Some of which, including miR-16-1, miR-16-2, have been shown to play a potential role in pulmonary edema. This dysregulation was statistically important, as it has been shown that miR-16-1 was overexpressed in infected cells as compared to non-infected ones.

### GO enrichment and pathways analysis

**Fig 6:**
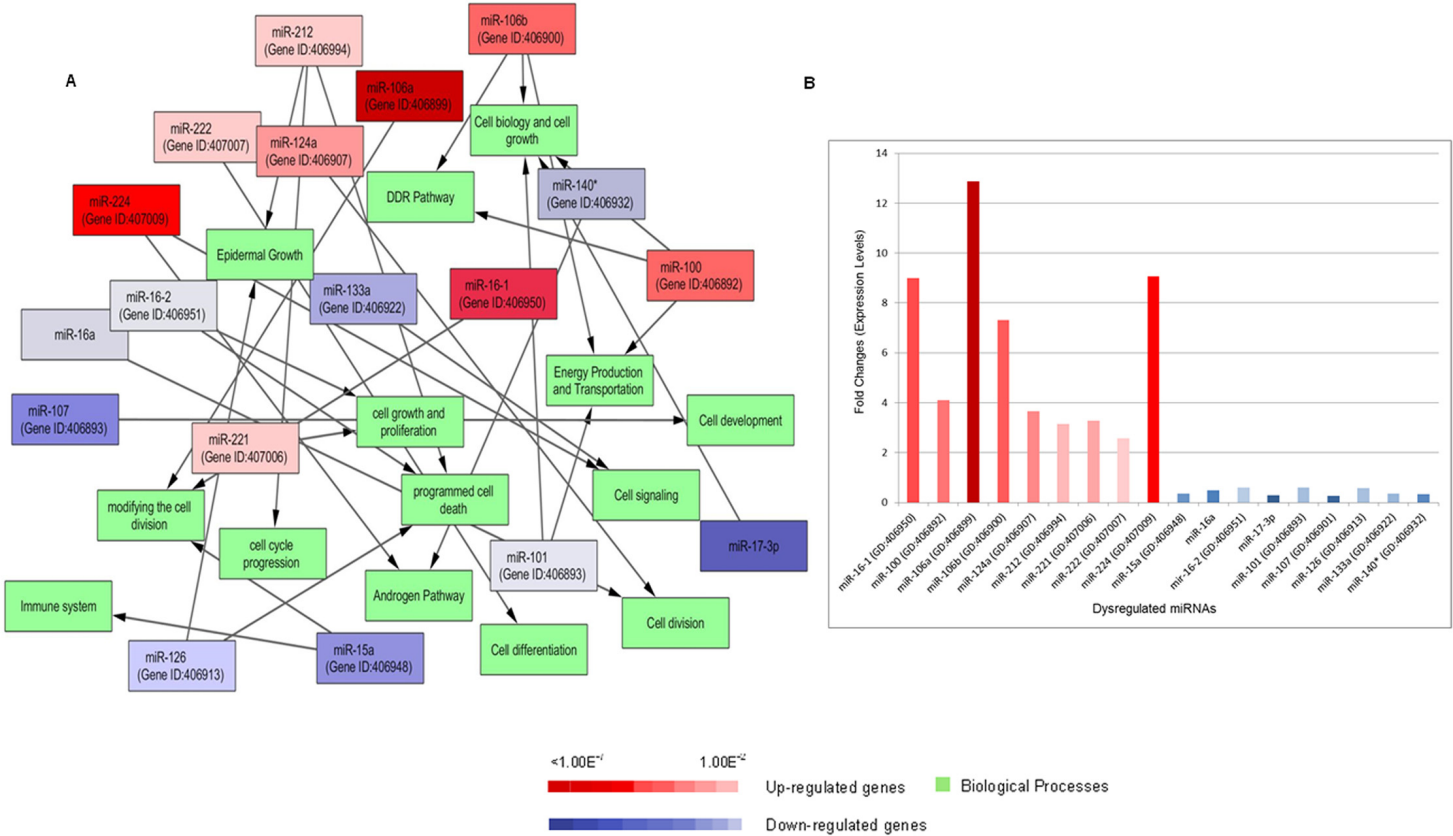
Pathways enrichment analysis. (A) Gene Ontology of biological processes inferred from dysregulated miRNA-targets in *Candida albicans* infected human respiratory epithelium. Red and blue color gradient intensity correlates with up- or down-regulation levels in comparison to normal cells. Green nodes are representing biological processes. Interactions were visualized as a network using Cytoscape visualization tool (B) Expression levels of these dysregulated miRNAs (up and down-regulated genes) in terms of fold changes.

In gene ontology (GO) enrichment analysis, it was found that infected epithelial cells up and down-regulate some biological processes. Several miRNAs expressed abnormally (>7-fold) as compared to the control. The interacting biological processes of these important genes were analyzed using the Cytoscape tool and effected pathways network have been shown in Fig 6A. The up and down regulated miRNAs (top selected according to Table 1) in infected respiratory epithelium affected the biological processes of pathways accordingly and fold changes expression levels of these dysregulated genes was analyzed (Fig 6B). It has been found that miR-16-1 and miR-15a are associated with E2F activity which is generated by pRB-E2F to regulate cell division process and migration [24]. The dysregulation of these genes is involved in developing the pulmonary edema [24]. Gene Ontology database showed that some genes (miR-100, 101, 106a) were associated with cell motility, energy production, active transportation, and DNA damage response (DDR pathway). The miR-16-2, 126, 212 are involved in several important and highly targeted genes and their pathways which have been highlighted including BCL2 (cell division, programme cell death and apoptosis) and EGFR (epidermal growth factor receptor). It was found that miR-140^*^ and miR-224 are linked to androgen pathway [25] and it was noted that the stimulation of these miRNAs transcription by androgen pathway is involved to suppress the tumor suppressor in lung cancer-1 gene [26].

## Discussion

The significant finding of this study is identification of a group of aberrant miRNA genes in respiratory epithelium infected by *Candida albicans*. We observed divergent expression patterns of miRNA genetic clusters which are involved in the regulation of important physiological pathways. How human cells respond to Candidal infection and how fungus acclimatize to host cell microenvironment remain largely unknown, which are critical for better understanding of host-pathogen interactions during *Candida albicans* infection.

Invasive candidiasis is the fourth most common bloodstream infection in USA [27] and this infection is getting worse in developing countries. The symptoms of this infection are not specific and it becomes fetal if not treated. Despite that some studies have been conducted on *Candida albicans* infections; our understanding about invasive candidiasis to modify the host cell’s response is still unclear. There are many pathogenic species which have been reported to alter cellular responses of their host through miRNAs which are now considered as master regulators of main cellular mechanisms [28]. Therefore, in this study, we hypothesized that invasive *Candida albicans* could change the miRNAs profile of human respiratory epithelium during infection. For this purpose, the purity of epithelial cells was analyzed by flow cytometry, indicating that the purity and viability of purified epithelial cells were over 85% and 94% respectively [29, 30].

This study has enabled us to identify aberrant miRNA gene expression in response to infection. We found that about 20% of the miRNAs were dysregulated upon infection. Some miRNAs, such as miR-16-1, 106a, 106b and 224, were significantly up-regulated [31] leading to uncontrolled proliferation.

It has been observed that endothelial cells behave differently to low and high densities of *C*. *albicans* [32]. Similarly it was found that *Candida* infection resulted in down-regulation of miR-17-3p and miR-107 genes that could lead to dysregulation of cell cycle, cell division and the signaling pathways related to inflammation and tumorigenesis. Some miRNAs was associated to control apoptotic and anti-apoptotic pathways. For example, inhibition of miR-212 activates the apoptosis level in epithelial cells [33]. In macrophages infected by *C*. *albicans*, Monk et al. in 2010 reported that miR-455, miR-125, miR-146 and miR-155 may play significant roles in regulating macrophage function following PRR stimulation [34]. Such observations were also reported by Kilic et al. in 2011 [35] who studied that sub-epithelial fibrosis in a mouse model can be blocked by switching off one of these factors and its over-expression has a direct effect on collagen expression in mouse lung fibrosis. Likewise, it was studied that aberrant expression of miR-212 in non-small cell lung cancer and in ovarian cancer cells induces apoptosis [36, 37]. Similarly, it was reported that dysregulation of miR-15a due to LMP1 stimulates cell cycle proliferation and predicts poor prognosis in nasal NK/T-cell lymphoma [38] and it has also been observed that this miRNA is frequently less expressed in non-small cell lung cancer tissues [26, 39]. MiR-155 miRNA is down-regulated in individuals infected with invasive candidiasis, and cytokine is a target protein that is involved in inflammatory signaling including IRAK1, Traf6 and Myd88 [34]. Interestingly miR-155 has been revealed to down-regulate DC-SIGN (a C-type lectin) that recognizes mannose containing glycoproteins expressed by different pathogens including fungi [40].

We also observed (as shown in Fig 6) that some miRNAs including miR-100, 106b, 222 was up-regulated during *Candida* infection. These miRNAs species were related to cell growth and development, active transportation and energy, and cell differentiation. The gene ontology enrichment analysis of these aberrant miRNAs showed variations in molecular functions, cellular and biological processes. Abnormal expression of miR-100 interfere the migration, proliferation pathway and causes gastric cancer [41].

This study would be a valuable addition for better understanding of mechanism of host responses after fungal infection, and additional investigation will be required to validate the dysregulated miRNAs in response to Candidal infection. Further studies are warranted to test these hypotheses, but our findings clearly demonstrate that dysregulated expression profiles of clusters of miRNAs are correlated with Candida infection.

## Conclusion

Our results indicate that miRNA profile of human respiratory epithelium is influenced by *Candida albicans*. Such alteration in miRNAs levels suggests dramatic host genetic responses to infectious diseases and will be helpful to better understand the treatment decisions.
